# Urantide improves atherosclerosis by controlling C-reactive protein, monocyte chemotactic protein-1 and transforming growth factor-β expression in rats

**DOI:** 10.3892/etm.2014.1654

**Published:** 2014-03-31

**Authors:** JUAN ZHAO, LI-DE XIE, CHENG-JUN SONG, XIAO-XIA MAO, HAI-RONG YU, QUAN-XIN YU, LI-QUN REN, YAN SHI, YA-QIN XIE, YING LI, SHA-SHA LIU, XIAO-HONG YANG

**Affiliations:** 1 School of Pharmaceutical Sciences, Jilin University, Changchun, Jilin 130021, P.R. China; 2 Department of Pathophysiology, Chengde Medical University, Chengde, Hebei 067000, P.R. China

**Keywords:** urantide, urotensin II, atherosclerosis, inflammatory cytokines, vascular smooth muscle cells

## Abstract

The aim of the present study was to investigate the effects of urantide on the expression status of C-reactive protein (CRP) and the inflammatory cytokines monocyte chemotactic protein (MCP)-1 and transforming growth factor (TGF)-β in the aortas of rats with atherosclerosis (AS), and to identify its underlying mechanisms. The effects of urantide in a rat model of AS and in cultured rat vascular smooth muscle cells (VSMCs) were analyzed via hematoxylin and eosin staining, immunohistochemical staining and ELISA. The results *in vivo* demonstrated that urantide downregulated the expression of inflammatory mediators CRP and MCP-1 and upregulated the expression of TGF-β. The results *in vitro* indicated that urantide inhibited the proliferation of VSMCs. In addition, urantide reduced the expression of CRP and downregulated the secretion of TGF-β in the culture supernatant. In conclusion, urantide ameliorated the arterial inflammatory damage that was observed in the AS rat model at the cell and tissue levels by controlling the expression of CRP and the inflammatory cytokines MCP-1 and TGF-β. Therefore, urantide may be a potential agent for the complementary treatment of AS.

## Introduction

Atherosclerosis (AS) is a common cardiovascular disease and a predominant cause of human fatalities (1); its mechanism and methods of treatment are an interesting field within medical research. AS exhibits the classical pathological features of a typical inflammatory response, including tissue degeneration/necrosis, monocyte/lymphocyte exudation and vascular smooth muscle cell (VSMC) fibroplasia. Furthermore, it displays specific manifestations, rather than common chronic inflammatory responses, including endothelial damage, lipid deposition, monocyte migration, calcification and thrombosis, which are involved in the formation of AS (2). These pathological changes are the complex responses of the vascular endothelium and arterial wall VSMCs to a variety of injuries, which involve numerous inflammatory cytokines (3,4). The inflammation hypothesis described by Ross (2) is that AS is a pathological process exhibiting chronic inflammatory response characteristics, in which inflammatory cytokines perform different functions, interact with one another and are not confined to AS lesions. C-reactive protein (CRP) is a biomarker of inflammation, which accelerates the inflammatory response of AS through various mechanisms (5). Monocyte chemotactic protein (MCP)-1 is a potent pro-inflammatory chemotactic factor for monocytes that is generated at sites of injury in AS rather than in the normal vessel wall (6). Transforming growth factor (TGF)-β is an anti-inflammatory cytokine that exhibits an anti-AS effect and is widely expressed in inflammatory cells and the atherosclerotic plaques of humans and rats (7). The balance between pro- and anti-inflammatory cytokines is the key factor that determines the occurrence of AS (8–11); therefore, the investigation of inflammation-associated molecules is significant to the treatment of AS.

Urotensin II (UII) is a somatostatin-like cyclic peptide, which was originally isolated from the urophysis of the goby fish (*Gillichthys mirabilis*) (3). UII binds to its receptor G protein-coupled receptor 14 (GPR14), forming a UII/urotensin receptor system, which initiates a series of biological effects, such as vasoconstriction (12,13). Urantide is a GPR14 antagonist peptide derived from human UII and is currently considered to be the predominant GPR14 antagonist, exhibiting an antagonistic effect that is 50–100-fold stronger compared with that of other chemical compounds (14–17). In the present study, urantide was administered to an AS rat model and applied to cultured VSMCs to investigate its effects on the expression of CRP and the inflammatory cytokines MCP-1 and TGF-β in the aorta. The contribution of UII to the formation and development of AS, in addition to the underlying mechanism of UII, were thereby investigated, thus providing an experimental basis for the use of urantide in the clinical prevention of AS.

## Materials and methods

### Reagents

Urantide was synthesized by Shanghai HD Biosciences Co., Ltd. (Shanghai, China). Fluvastatin (Flu; 40 mg/box) was purchased from Novartis Pharma Inc. (Beijing, China), Dulbecco’s modified Eagle’s medium (DMEM) and UII were obtained from Gibco-BRL (Carlsbad, CA, USA). Fetal bovine serum (FBS) was purchased from Tianjin Haoyang Biological Manufacture Co., Ltd. (Tianjin, China), anti-α-smooth muscle actin (SMA) monoclonal antibody was purchased from Beijing Biosynthesis Biotech Co., Ltd. (Beijing, China) and anti-rat CRP, MCP-1 and TGF-β monoclonal antibodies were purchased from Santa Cruz Biotechnology, Inc. (Santa Cruz, CA, USA). Horseradish peroxidase-labeled secondary antibodies were obtained from Wuhan Boster Biological Technology, Ltd. (Wuhan, China) and a streptavidin-biotin complex (S-ABC) immunohistochemical kit and 3,3′-diaminobenzidine (DAB) coloring kit were purchased from Maixin Biological Technology Development Co., Ltd (Fuzhou, China).

### Rat modeling and animal treatment

A total of 120 healthy male Wistar rats (weight, 180–200 g) were fed in the Animal Center of Bethune Medical School of Jilin University (Changchun, China; license no. SCXK[JI]-2009-0004) and the experimental procedures were approved by the Jilin University Ethics Committee. For preparation of the rat model, 100 rats were fed a high-fat forage and simultaneously underwent an intraperitoneal injection of 70 U/kg vitamin D3 for three consecutive days. The basic diet composed of 73.5 wt.% corn, 20 wt.% wheat bran, 5 wt.% fish meal, 1 wt.% flour and 0.5 wt.% salt, was supplied by Jilin University Laboratory Animal Center (Changchun, Jilin, China). A high-fat diet was composed of 80.8 wt.% basic diet, 3.5 wt.% cholesterol (Changchun Baiao Biotech Inc., Changchun, Jilin, China), 10 wt.% lard (WalMart, Changchun, Jilin, China), 0.2 wt.% propylthiouracil (Fosun Pharmaceutical Co., Ltd., Shanghai, China), 0.5 wt.% sodium cholate (Gibco-BRL, Carlsbad, CA, USA) and 5 wt.% sugar (WalMart). Six weeks subsequently, hematoxylin and eosin (H&E) staining was performed to enable observation of the morphological changes that had occurred in the aortas of the rats. Following AS modeling, the rats were randomized into four groups: Normal control (NC), n=20; AS model control (ACS), n=20; Flu, n=20; and urantide groups, n=60 (divided into three subgroups of 3, 7 and 14 days). The NC and ASC groups were injected with 0.9% NaCl solution (dose, 30 μg/kg) into the tail vein, once a day for 14 days. The Flu group were administered Flu intragastrically (dose, 5 μg/kg) once a day for 14 days. The urantide groups were injected with urantide (dose, 30 μg/kg) into the tail vein once a day for 3, 7 or 14 days.

### Detection of blood calcium (Ca^2+^) and fat levels

The rats were fasted overnight and injected intraperitoneally with 0.3% pentobarbital sodium (dose, 30 mg/kg) for anesthetization. The aorta pectoralis was separated and the arterial blood was collected using a 5-ml syringe. The blood serum was separated by centrifugation at 1,000 × g for 15 min and was allocated to Eppendorf tubes (Eppendorf China Ltd., Shanghai, China) and stored at −20°C. A FACA 401 automatic biochemistry analyzer (Labomed, Inc., Culver City, CA, USA) was used to examine the levels of Ca^2+^, triglycerides (TG), total cholesterol (TC), high density lipoprotein (HDL) and low density lipoprotein (LDL) in the blood.

### CRP, MCP-1 and TGF-β expression in the aortic tissues

Immunohistochemical staining was performed using a S-ABC immunohistochemical kit, according to the manufacturer’s instructions, to detect the protein expression in the paraffin-embedded sections of the rat aortic tissue. Anti-CRP, MCP-1 and TGF-β monoclonal antibodies were diluted (1:100) and DAB was used as the chromogen for 5 min at room temperature. The sections were counterstained and sealed using a neutral resin. Ten random microscopic fields (magnification, ×400) of each section were observed and the average grayscale of the immunohistochemical positive signal was analyzed using ImageJ software (National Institutes of Health, Bethesda, MA, USA).

### Isolation and identification of primary VSMCs

The procedure for the isolation of VSMCs was conducted in accordance with the methods of a previous study (18). Briefly, Wistar rats were anesthetized by inhalation of ether and were sacrificed via dislocation. The aorta was immediately removed, placed in 75% alcohol, dissected into sections (length, 3 cm) and subsequently rinsed using phosphate-buffered saline (0.2 mol/l, pH 7.2). The tunica externa (the external third of the vessel wall thickness) contains fibroblasts that were carefully peeled using forceps. The residual vessels were cut longitudinally and the tunica interna was scraped off using a lancet, leaving the tunica media. The tunica media was washed using DMEM, cut into sections (1 mm^3^) and cultured in a culture flask with DMEM containing 10% FBS, at 37°C in a CO_2_ incubator (Heraeus CO_2_ incubator; Thermo Fisher Scientific Inc., Hudson, NH, USA). The microscopic morphology was observed using routine H&E staining and cell identification of SMA was performed using an S-ABC immunohistochemical kit. The α-SMA antibody was diluted (1:100) and DAB served as the chromogen. The number of positive cells was counted under a microscope (magnification, ×100) and the percentage was calculated.

### Detection of inflammatory mediators in the culture supernatant

VSMCs were suspended and seeded in each well of a 24-well plate at a density of 1×10^4^ cells/ml and a volume of 2 ml/well. As the cells reached 80% confluence, DMEM containing 0.5% serum was refreshed and incubated for 24 h. The VSMCs were subsequently treated in four separate groups: i) The normal cell control (CC) group was cultured with DMEM containing 10% FBS; ii) the urotensin control (UC) group was treated with UII using a 10^−8^ mol/l final concentration; iii) the Flu + UII group was treated with 10^−7^ mol/l Flu and 10^−8^ mol/l UII; and iv) the urantide + UII group was treated with between 10^−10^ and 10^−6^ mol/l urantide and 10^−8^ mol/l UII. After a further 48 h incubation, the culture supernatants were collected and an ELISA was performed to assess the levels of CRP, MCP-1 and TGF-β. Briefly, 0.5 ml of supernatants was absorbed to microplates (Thermo Fisher Nunc Inc., Roskilde, Denmark) overnight. Then 0.2 ml of anti-rat CRP, MCP-1 and TGF-β antibodies were added, respectively, and incubated 2 h at room temperature. Then 0.2 ml of HPR-labeled secondary antibody was added to incubate 1 h at room temperature. DAB was used for color development. The absorbance value of each well was detected at 450 nm using a Multiskan Microplate reader (Thermo Fisher Scientific Inc., Hudson, NH, USA).

### Statistical analysis

Data are expressed as the mean ± standard deviation. Significant differences were examined using SPSS software, version 13.0 (SPSS Inc., Chicago, IL, USA). Analysis of variance was used to analyze the interclass differences and the least significant difference method was used for multiple comparisons of the ad hoc tests. P<0.01 was considered to indicate a statistically significant difference.

## Results

### Blood calcium and fat levels

Table I shows the results of the blood analysis. Levels of blood Ca^2+^ and lipids (TG, TC, HDL and LDL) in the ASC group were increased significantly compared with those in the NC group (P<0.01), but were significantly reduced in the Flu group compared with those in the ASC group (P<0.05 or P<0.01). A gradual reduction observed over time in the urantide groups; at day 14, the levels were comparable to those in the Flu group.

### Arterial morphology

**Figure 1:**
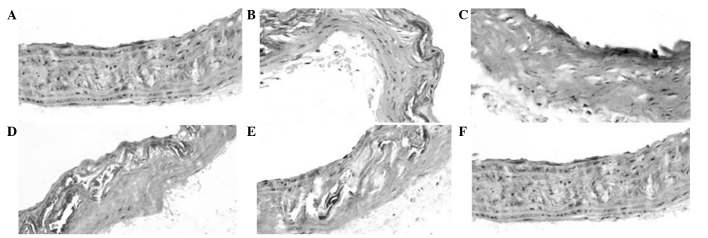
Microscopic morphology of the aortic tissue of rats in the various groups (hematoxylin and eosin stained; magnification, ×200). (A) Normal control group. (B) Atherosclerosis control group. (C) Fluvastatin group. (D–F) Urantide subgroups, at 3, 7 and 14 days, respectively.

Fig. 1 demonstrates the microscopic morphology of the aortic tissue sections, which were detected using H&E staining. In the NC group, the tunica intima, media and externa of the rat aorta exhibited clear borders, the vascular integrity of the endothelium was maintained, spindle smooth muscle cells were identified in the tunica media, the elastic fiber layer exhibited a clear structure and integral rings, and the connective tissues of the tunica externa were loose. In the ASC group, the aortic tunica intima exhibited typical pathological changes, including apparent calcification and infiltration by inflammatory cells, VSMC hyperplasia and foam-like accumulation, degeneration, breakage and disintegration of the elastic fibers, in addition to atrophia of the tunica media. However, in the Flu group, the pathological changes were ameliorated compared with those in the ASC group, calcification was not observed and there was only marginal inflammatory cell infiltration, slight VSMC hyperplasia and a small number of foam-like cells. In the rats treated with urantide, the pathological changes were gradually attenuated as the treatment time increased, and were comparable to those in the Flu group by day 14.

### CRP, MCP-1 and TGF-β expression in the aortic tissues

**Figure 2:**
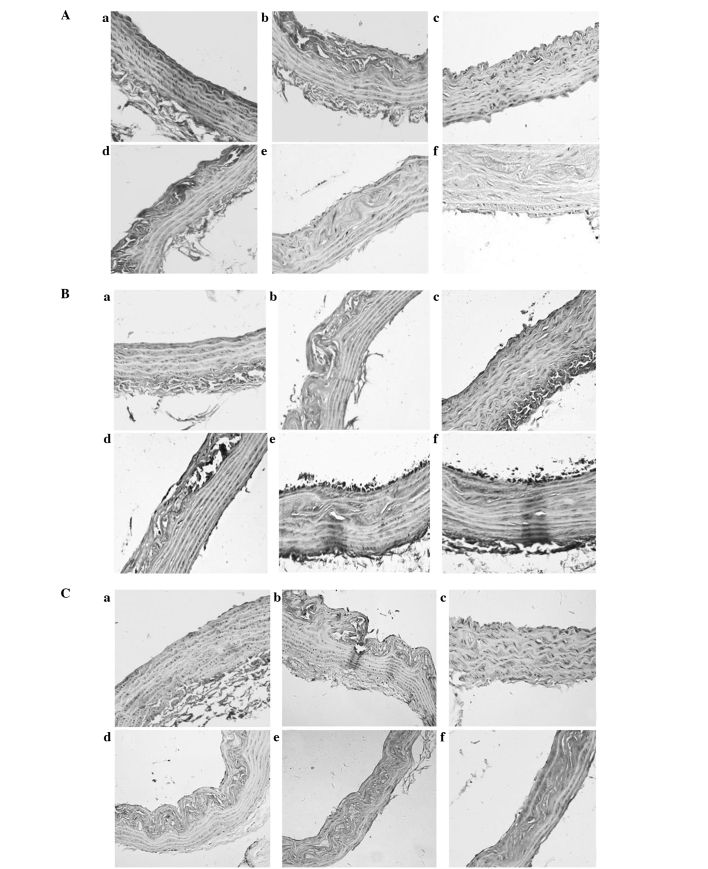
Immunohistochemical staining of (A) C-reactive protein, (B) monocyte chemotactic protein-1 and (C) transforming growth factor-β in the aortic tunica intima and tunica media observed in the various groups (magnification, ×400); (a) normal control group; (b) atherosclerosis control group; (c) fluvastatin group; (d–f) urantide groups at 3, 7 and 14 days.

Immunohistochemical staining was used to detect CRP, MCP-1 and TGF-β expression in the aortic tissue of the rats (Fig. 2). In the NC group, some positive expression of CRP and TGF-β was apparent in the tunica intima and tunica media; however, there was no expression of MCP-1 observed in the tunica intima or media. In the ASC group, positive expression of CRP in the tunica intima, tunica media and the AS plaques significantly increased compared with that observed in the NC group. The positive expression of MCP-1 was somewhat apparent in the AS plaques and the expression of TGF-β increased markedly in the AS plaques compared with that of the NC group. In the Flu group and the urantide subgroups, compared with the ASC group, the CRP and MCP-1 expression levels were reduced and TGF-β expression was upregulated in the AS plaques. Table II indicates the results of the semi-quantitative analysis that was conducted using ImageJ software. The effect of urantide on the CRP and MCP-1 factors was time-dependent and TGF-β expression peaked 7 days after urantide was first injected.

### Morphology and identification of VSMCs

**Figure 3:**
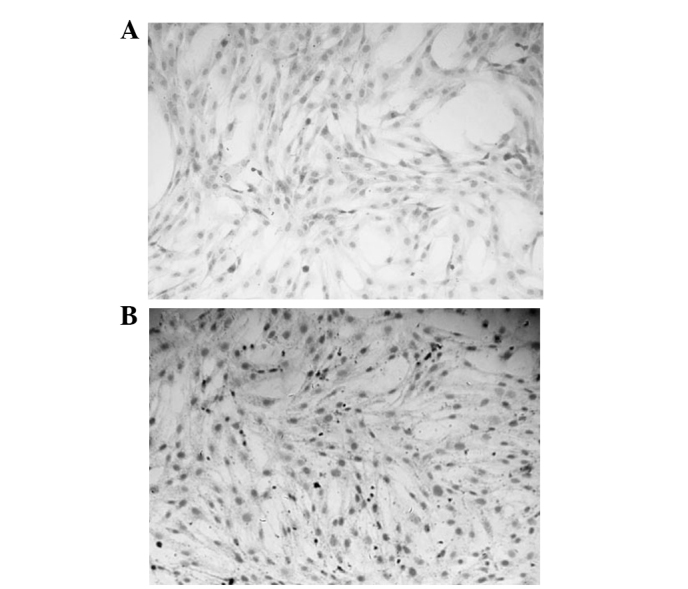
(A) Hematoxylin and eosin stained cultured vascular smooth muscle cells (VSMCs; magnification, ×100). (B) α-Smooth muscle actin antibody stained VSMCs (streptavidin-biotin complex immunohistochemical staining, magnification, ×100).

The microscopic passaged VSMCs that underwent H&E staining (Fig. 3A) were rhombic, triangular, ribbon- or star-shaped and the nuclei of the cells were predominantly oval-shaped. The cytoplasms were pink-stained and the nuclei were purple blue. Following S-ABC immunohistochemical staining of α-SMA in cultured VSMCs, the cell cytoplasms were stained brown and the nuclei were not stained; the fraction of positively stained cells was >98% (Fig. 3B).

### CRP, MCP-1 and TGF-β expression in cell culture supernatant

The levels of CRP, MCP-1 and TGF-β in the supernatant of cultured VSMCs were detected by ELISA (Table III). In the UC group treated with UII only, the CRP and TGF-β levels were significantly increased compared with the levels observed in the CC group (P<0.01); however, the MCP-1 levels did not change. In the Flu + UII group and each of the urantide + UII subgroups, the CRP and TGF-β levels were significantly reduced, compared with those in the UC group (P<0.01); the MCP-1 levels did not change. Thus, the effects of urantide on CRP and TGF-β levels were observed to be concentration-dependent. Moreover, between 10^−8^ and 10^−6^ mol/l urantide reduced the expression of CRP to levels comparable with those in the CC group and 10^−6^ mol/l urantide was identified as the optimal concentration for the downregulation of CRP.

## Discussion

Urantide is an antagonist of the UII receptor, GPR14, which is able to competitively bind to GPR14, thus antagonizing aortal contraction in rats (12,13). In addition, urantide may have significant effects on the expression of certain inflammatory mediators. This is significant when investigating the mechanism of UII and the interactions of inflammatory mediators in AS.

In the present study, analyses *in vivo* and *in vitro* were performed to observe how urantide influenced the expression of CRP and the inflammatory cytokines MCP-1 and TGF-β in the aorta and artery-derived VSMCs of rats with AS. *In vivo* daily administration of urantide for three days resulted in reduced blood TG, TC, HDL and LDL levels in rat aortic tissue, in addition to the downregulation of the expression of the inflammatory mediators CRP and MCP-1, thus improving the symptoms of AS. These effects were identified to be time-dependent. The use of UII *in vitro* increased the proliferation of rat VSMCs and elevated the content of CRP in the VSMC culture supernatant. Following the use of urantide *in vitro*, CRP and TGF-β expression levels were reduced. These findings indicate that UII accelerated the inflammatory arterial damage during the formation and development of AS, while urantide, as a UII receptor antagonist, reduced the inflammatory arterial damage and ameliorated the symptoms of AS. Furthermore, one of the mechanisms of urantide action was the control of the expression of inflammatory mediators, which included downregulation of CRP and MCP-1 and upregulation of TGF-β *in vivo*.

Furthermore, MCP-1 levels *in vivo* increased in the AS rat models and decreased as a result of urantide administration, however, the MCP-1 levels *in vitro* did not change following the use of UII or urantide. This indicated that AS involves certain UII-independent mechanisms or pathways *in vivo* that affect MCP-1 expression but do not occur *in vitro*.

Urantide is a high-performance UII receptor antagonist, which has the potential to be a complementary treatment for AS, particularly when the pathophysiological effects of urantide on cardiovascular diseases have been clarified.

## Figures and Tables

**Table I:** Blood Ca^2+^, TG, TC, HDL and LDL concentrations in rats (mean ± standard deviation; n=5).

Group	Ca^2+^ (mmol/l)	TG (mmol/l)	TC (mmol/l)	HDL (mmol/l)	LDL (mmol/l)
NC	2.62±0.06	0.03±0.01	1.20±0.02	0.57±0.04	0.16±0.01
ASC	3.82±0.01^a^	1.74±0.08^a^	17.61±0.08^a^	2.19±0.05^a^	16.05±0.15^a^
Flu	3.12±0.01^a^,^c^	0.74±0.05^a^,^b^	12.45±0.02^a^,^c^	1.27±0.00^a^,^c^	11.95±0.06^a^,^c^
Urantide					
3 days	3.57±0.00^a^,^c^	1.04±0.00^a^,^c^	17.03±0.01^a^,^c^	1.98±0.00^a^,^c^	15.16±0.01^a^,^c^
7 days	3.48±0.01^a^,^c^	0.72±0.14^a^,^b^	15.87±0.00^a^,^c^	1.81±0.02^a^,^c^	14.20±0.01^a^,^c^
14 days	3.43±0.39^a^,^c^	0.43±0.04^a^,^c^	12.83±0.06^a^,^c^	1.48±0.06^a^,^c^	10.16±0.05^a^,^c^

Analysis of variance was used to compare intergroup differences, the least significant difference method was performed for the ad hoc analyses.

a P<0.01 vs. the NC group;

b P<0.05 and

c P<0.01 vs. the ASC group.

TG, triglycerides; TC, total cholesterol; HDL, high density lipoproteins; LDL, low density lipoproteins; NC, normal control; ASC, atherosclerosis control; Flu, fluvastatin.

**Table II:** Expression of CRP, MCP-1 and TGF-β in aortic tunica intima and tunica media (grayscale values; mean ± standard deviation; n=5).

Group	CRP	MCP-1	TGF-β
NC	10.04±0.02	1.60±0.01	30.36±0.03
ASC	101.36±0.11^a^	41.70±0.01^a^	46.64±0.11^a^
Flu	12.65±0.71^a^,^c^	3.83±0.01^a^,^c^	50.85±0.01^a^,^c^
Urantide			
3 days	83.90±0.59^a^,^c^	2.56±0.05^a^,^c^	74.96±0.05^a^,^c^
7 days	54.67±0.48^a^,^c^	1.81±0.19^c^	94.81±0.01^a^,^c^
14 days	31.22±0.11^a^,^c^	1.83±0.08^c^	47.05±0.51^a^,^b^

Analysis of variance was used to compare intergroup differences, the least significant difference method was performed for the ad hoc analyses.

a P<0.01 vs. the NC group;

b P<0.05 and

c P<0.01 vs. the ASC group.

CRP, C-reactive protein; MCP-1, monocyte chemotactic protein-1; TGF-β, transforming growth factor-β; NC, normal control; ASC, atherosclerosis control; Flu, fluvastatin.

**Table III:** CRP, MCP-1 and TGF-β content in vascular smooth muscle cell culture supernatant (optical density; mean ± standard deviation; n=5).

Group	CRP	MCP-1	TGF-β
CC	1.63±0.18	2.22±0.03	0.94±0.01
UC	2.20±0.07^b^	2.31±0.07	1.67±0.02^b^,^d^
Flu + UII	1.74±0.03^d^	2.26±0.04	1.11±0.04^d^
Urantide + UII (mol/l)			
10^−10^	1.90±0.02^b^,^d^	2.28±0.02	1.07±0.01^d^
10^−9^	1.84±0.08^b^,^d^	2.28±0.02	1.13±0.01^d^
10^−8^	1.69±0.02^d^	2.26±0.01	1.14±0.00^d^
10^−7^	1.68±0.02^d^	2.24±0.02	1.22±0.01^b^,^d^
10^−6^	1.64±0.07^d^	2.23±0.02	1.32±0.04^b^,^d^

Analysis of variance was used to compare intergroup differences, the least significant difference method was performed for the ad hoc analyses.

a P<0.05 and

b P<0.01 vs. CC group;

c P<0.05 and

d P<0.01 vs. UC group.

CRP, C-reactive protein; MCP-1, monocyte chemotactic protein-1; TGF-β, transforming growth factor-β; CC, cell control; UC, urotensin II control.
